# Phenotypical Comparison between Environmental and Clinical *Acinetobacter baumannii* Strains Isolated from an Intensive Care Unit

**DOI:** 10.21315/mjms2023.30.4.8

**Published:** 2023-08-24

**Authors:** Saliha Lydia Boulesnam, Fella Hamaidi-Chergui, Mounia Benamara, Sihem Azrou

**Affiliations:** 1 Biotechnology, Environment and Health Laboratory, Biology Department, Blida 1 University, Algeria; 2 Central Laboratory, Blida EPH, Algeria; 3 Microbiology Laboratory, Faculty of Pharmacy of Algiers, Algeria; 4 Central Laboratory, Beni Messous EPH, Algeria

**Keywords:** Acinetobacter baumannii, multidrug-resistant, biofilm, nosocomial infections

## Abstract

**Background:**

*Acinetobacter baumannii* (*A. baumannii*) causes a variety of nosocomial infections that mainly affect critically ill patients in intensive care units (ICUs). The objective of this study was to assess the prevalence of *A. baumannii* in the ICU environment and evaluate the antibiotic resistance and biofilm formation ability of the environmental isolates compared to those isolated from ICU patients simultaneously.

**Methods:**

A total of 166 non-duplicate ICU samples (80 environmental and 86 clinical) were collected between January 2019 and January 2020. Antimicrobial susceptibility detection was determined using the disc diffusion method, and the strains were evaluated for the minimum inhibitory concentration (MIC) of imipenem (IMP) using broth microdilution or metallo-β-lactamase (MBL) detection according to the Clinical and Laboratory Standards Institute (CLSI) guidelines. The isolates’ capacity to produce biofilms was evaluated using the tube method and the crystal violet microtitre plate-based method.

**Results:**

*A. baumannii* was identified in 25 (31.25%) environmental and 30 (34.88%) clinical samples, and beds were the most infected (60%). Both types of isolate demonstrated a rate surpassing 80% resistance to the tested antibiotics. Phenotypically, the environmental and clinical strains were found to be MBL producers. Fourteen environmental (56%) and 15 clinical (50%) strains were found to be moderate biofilm producers, indicating that each isolate has a high biofilm-forming capacity.

**Conclusion:**

These results show that the spread of multidrug-resistant (MDR) *A. baumannii* in an ICU setting emphasises the necessity of disinfecting and cleaning medical devices and surfaces to prevent and restrict cross-transmission. Intensive surveillance and infection control methods are also of paramount importance.

## Introduction

The genus *Acinetobacter* is an oxidase-negative coccobacillus with over 40 genospecies, and *Acinetobacter baumannii (A. baumannii*) is considered the most incriminating of a wide range of infection syndromes. It accounts for 2%–10% of all Gram-negative hospital infections, with most occurring in intensive care units (ICUs) (1, 2). It can cause a variety of nosocomial infections, including meningitis, urinary tract, bloodstream and wound infections and ventilator-associated pneumonia (3). The multidrug-resistant *A. baumannii* (MDR-AB) has emerged as a major global health concern (4), because of the increased use of antimicrobials in recent years, this bacterium is continuously exposed to antibiotic selection pressure, which encourages the acquisition of the resistance element (5). *A. baumannii* has a range of pathways for antibiotic resistance, as well as the production of extended spectrum beta-lactamase enzymes, active drug efflux pumps, modification enzymes and changed binding sites, the main cause identified as promoting carbapenem resistance in *A. baumannii* is the production of carbapenemases, such as class B metallo-β-lactamase (MBL) (6, 7).

Hospital surfaces play an important role in nosocomial infections. In ICUs, *A. baumannii* infection can be related to a lack of environmental surface cleaning and the continued use of medical equipment (8). The formation of biofilms on biotic and abiotic surfaces is a successful method to increase bacterial survival. However, *A. baumannii* can survive in a hostile hospital environment for long periods, which makes infections difficult to control (9, 10).

In view of this, the present study aimed to determine the prevalence of *Acinetobacter* infections in clinical setting in a hospital sited in southwest of Algiers (Algeria), as well as the profile of multidrug resistance (MDR) in *A. baumannii* isolated from the environment and from clinical samples, as well as its potential for biofilm formation.

## Methods

### Sample Collection

Eighty non-duplicate environmental samples were taken from different surfaces and instruments (beds, tables, serum stands, trolleys, doors, scopes, etc.) in the ICU of a public hospital in south-west Algiers for 1 year. Eighty-six non-duplicate clinical isolates were collected from a variety of clinical samples, including surgical wounds, blood, urine, wounds and catheter tips, as part of a routine hospital laboratory.

### Sample Identification

According to Baumann (11), swabs obtained from the hospital settings were put in 5mL brain heart infusion broth (BHI) and incubated for 24 h. All samples were cultured on Hektoen and blood agar plates under strict aseptic conditions, and then incubated at 37 °C for 24 h–48 h. Colonies were initially identified morphologically and on the basis Gram-staining, oxidase and triple sugar iron assays, then they were screened by their biochemical profiles.

### Antimicrobial Susceptibility Testing

The antimicrobial sensitivity of each isolate was determined using the disc diffusion method. The antimicrobials for different classes of antimicrobial frequently prescribed for *A. baumannii* infections according to the Clinical and Laboratory Standards Institute (CLSI) guidelines were ceftazidime (CAZ) (30 μg), imipenem (IMP) (10 μg), meropenem (30 μg) (MEM), levofloxacin (LEV) (5 μg), ticarcillin + clavulanic acid (TCC) (75/10 μg), tobramycin (TM) (10 μg), gentamicin (GM) (15 μg), amikacin (AN) (30 mg), piperacillin (PIP) (100 μg) and sulfamethoxazole/trimethoprim (SXT) (23.75/1.25 μg).

For carbapenem-resistant isolates identified by the disk diffusion test, the minimum inhibitory concentrations (MICs) of IMP were also determined by agar dilution and interpretation was made according to the CLSI guidelines. As reference strains for antibiotic quality control, *Escherichia coli ATCC 25922* and *Pseudomonas aeruginosa ATCC 27853* were used in all the antimicrobial susceptibility analyses.

### Phenotypic Detection of Carbapenemase and Metallo-β-Lactamase Isolates

The IMP-ethylene diamine tetra-acetic acid (EDTA) combined disk was used to determine MBL production by the IMP-resistant *A. baumannii* isolates according to the CLSI guidelines (12). In this disc potentiation test strains are cultured on Mueller Hinton agar, an IMP disc (10 μg) is placed adjacent (20 mm apart) to an IMP + EDTA disc that has already been prepared (10 μL of 0.1 M EDTA solution is put on an IMP disc). Negative controls were EDTA only discs. The test was considered positive if the inhibition zone around the disc with IMP-EDTA was enhanced by greater than or equal to 5 mm compared to the IMP disk alone.

### Biofilm Formation Tests

All the isolates were screened for their ability to produce biofilm using the tube method, as described by Christensen et al. (13), which consists of qualitative detection of extracellular polymeric substances. In this assay a visible coating around the inner wall on bottom of the tube was viewed as an indication of biofilm development. The results were scored visually as 0 (absent), + (weak/moderate) and + + + (strong). For quantitative biofilm detection beyond isolates, a microtitre plate-based method was used. In this test, a loop-full of the bacterium was inoculated into 5 mL trypticase soja broth supplemented with 1% glucose, and it was left to incubate for 24 h at 37 °C. After, the inoculum was diluted 1:100 with a new medium, then 0.2 mL of the diluted culture was introduced into each well of sterile polystyrene 96 well flat bottom tissue culture plates, then incubated at 37 °C for 24 h. After incubation, the plate’s contents were carefully scraped off and washed four times in phosphate-buffered saline (pH 7.2) to remove any floating bacteria. With 2% sodium acetate, biofilms produced by attaching sessile bacteria are fixed, then the plates were dyed for 15 min at a room temperature with 0.1% crystal violet then they were dried after being washed four times in deionised water to remove any residual stains.

With a micro ELISA auto reader set to optical density (OD) 630 nm, the OD of stained adhering bacteria was determined. To deduct the average from all test results, the OD values from the fixative dye, and sterile media were also averaged. Three separate runs of the tests were performed in triplicate. Based on the OD values of the different isolates, bacterial adherence was classified. Mean OD values between 0.120 and 0.240, 0.20 to 0.240 and > 0.240, respectively, were categorised as non- or weakly, moderate and strong biofilm adherence (13, 14).

### Statistical Analysis

Descriptive statistical analysis was performed using Microsoft Excel 2016. The data on antimicrobial susceptibility were analysed and compared using Student’s *t*-test and χ^2^ tests in the search for eventual links between response and sample types. The Mann-Whitney U test was used to compare the OD 570 values for both types of isolate. The data analysis was conducted using Statistica 64, version 10.0 (Stat Soft Inc., Tulsa, OK, USA). The significance level for all statistical tests was set at *P* < 0.05.

## Results

### Strain Identification and Distribution

During the study period, *A. baumannii* was found in 25/80 environmental swabs (31.25%), with the distribution varying depending on the sample site. Of the 86 samples obtained from non-duplicate clinical samples, 30 samples (34.88%) were confirmed as *A. baumannii*. Due to serious head trauma and cerebrovascular accidents that required extended antibiotic therapy, most patients admitted to the ICU underwent mechanical ventilation.

The majority of *A. baumannii* isolates were obtained from the surgical wound (73.33%) and from beds (60%) (Table 1).

### Antimicrobial Susceptibility Testing

**Figure 1:**
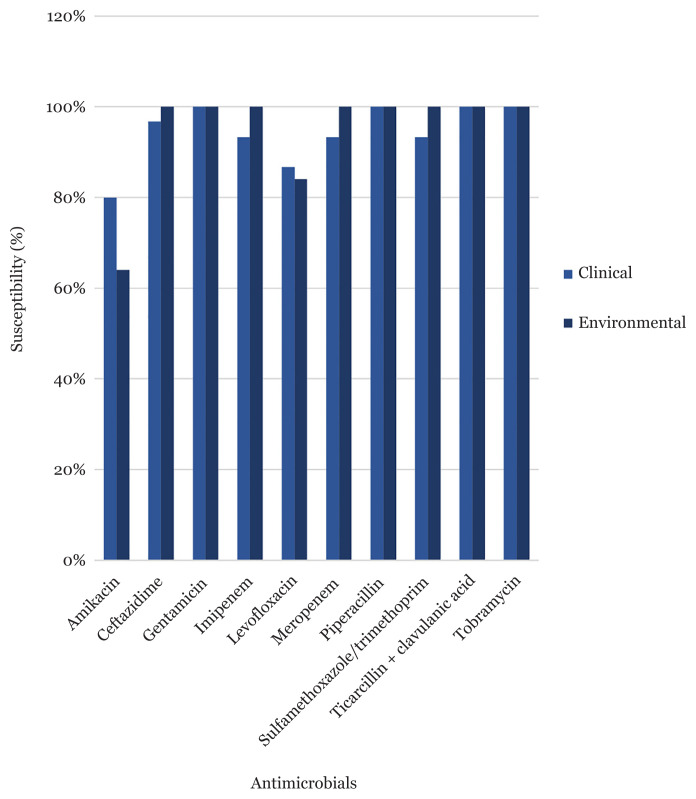
Antimicrobial susceptibility of environmental and clinical *A. baumannii*

The antibiotic susceptibility pattern of *A. baumannii* isolated from clinical and environmental samples was 100%. They demonstrated an antibiotic resistance rate of greater than 80% to the majority of the examined antibiotics (9 out of 10), as shown in Figure 1, as results all strains are considered as multidrug resistant. We observed a majority of MIC IMP was greater than 64 μg/mL, clinical and environmental strain resistance was, respectively, 27 (90%) and 25 (100%).

### Antimicrobial Profile Comparison of Environmental and Clinical Isolates

The distribution of the various resistotypes detected among *A. baumannii* isolates is presented in Table 2. A total of 55 (I–VI) resistotypes were identified, and the most predominant profiles were found to be resistotype I (resistant to AMK/CAZ/GEN/IMP/LVX/MEM/PIP/SXT/TCC/TIC; 73% and 64%) and II (resistant to CAZ/GEN/IMP/LVX/MEM/PIP/SXT/TCC/TIC; 10% and 46%).

According to the statistical interpretation (Table 3), no significant difference was found between the number of clinical and environmental strains in terms of resistance to the different antibiotics (*P* > 0.05). The chi-squared test showed a very highly significant relationship between clinical and environmental strains and their resistance profile (*P* = 0.0006).

### Phenotypic Detection of Carbapenemase and Metallo-β-Lactamase Isolates and Biofilm-forming Potential

**Figure 2:**
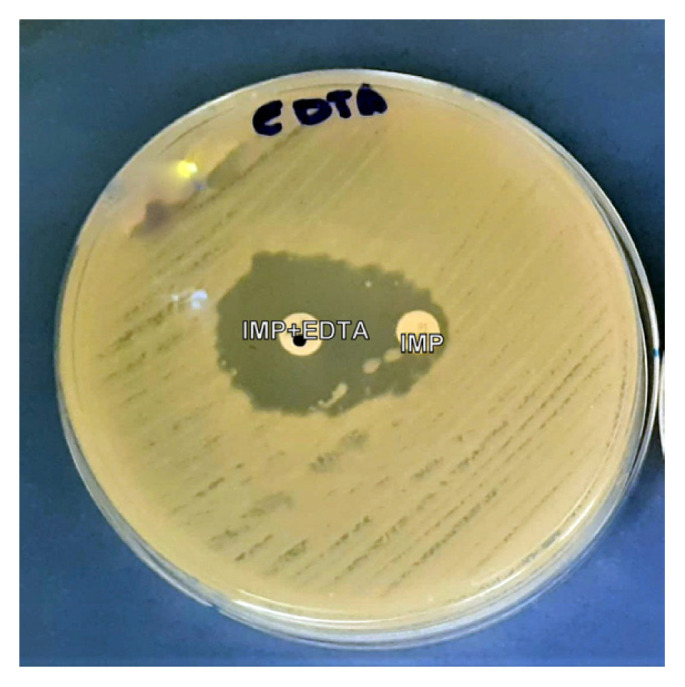
IMP-EDTA disk method for MBL producing environmental and clinical isolates

**Figure 3:**
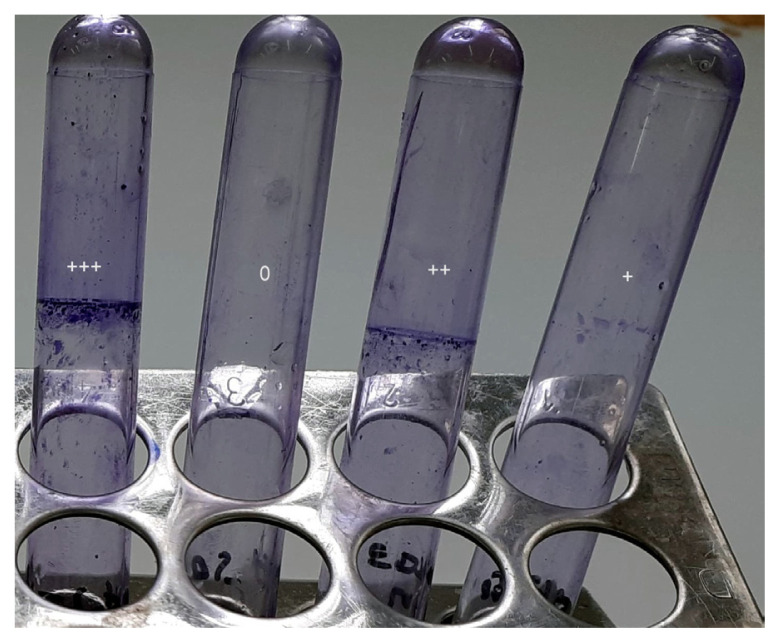
Detection of biofilm forming *A. baumannii* by tube method Notes: 0: none; +: weak; ++: moderate; +++: strong

The EDTA test was positive for the vast majority of clinical (93.33%) and environmental strains (100%) (Figure 2). The tube method was used to determine the ability of the strains to form biofilm (Figure 3). The results were confirmed by measuring the optical density of each strain.

For environmental isolates, 14 (56%) were moderate producers, 9 (36%) were strong biofilm producers and 2 (8%) were non-biofilm producers. For clinical isolates, 15 (50%) were moderate producers, 13 (43.4%) were strong biofilm producers and 2 (6.6%) were non-biofilm producers (Figure 3). A Mann-Whitney U test did not reveal significant differences (*P* = 0.14) in biofilm production between the two types of isolate.

## Discussion

*A. baumannii* is a frequent opportunistic pathogen that undoubtedly causes a range of nosocomial infections, mostly affecting patients who have been intubated and who have many intravenous lines, monitoring devices, surgical drains or urine catheters in place (15, 16). In this study, diverse sites were contaminated with *A. baumannii*, most isolates were from the surgical wound (73.33%) and beds (60%), which might be considered a probable environmental reservoir of *A. baumannii*, emphasising the concept that many areas in ICUs must be maintained with extreme caution (17). According to Hess et al. (18) patients, healthcare workers and hospital equipment spread the major of strains incriminated in hospital outbreaks.

In our analysis, *A. baumannii* proved to be multidrug resistant, all environmental and clinical isolates were resistant to most commonly used antibiotics such as aminoglycosides, cephalosporin, carbapenem and quinolones, particularly a low susceptibility to carbapenem (IMP and meropenem), likewise Bakour et al. (19) and Touati et al. (20) study found the same high frequency of antibiotic-resistant *A. baumannii* isolated from hospital surfaces, settings and clinical samples in an Algerian hospital. Additionally, Markogiannakis et al. (21) also reported that *A. baumannii* isolated from clinical and inanimate surfaces were resistant to: tobramycin, colistin, gentamicin, and meropenem causing sepsis outbreaks in a trauma ICU in Greece.

In our study, MICs IMP for clinical and environmental strains show a high rate of resistance, 90% and 100%, respectively, that was greater than 64 μg/mL. Further, a study conducted by Mesli et al. (22) in three different hospitals situated in north-western Algeria, reported that in total of 100 clinical and 13 hospital environment isolates, 106 *A. baumannii* was found, where 80 were IMP-resistant strains with MIC ranging from 64 mg/mL to 512 mg/mL.

Resistance profile comparison defined that there is a strong similarity between environmental and clinical isolates, which may indicate that the ICU environment is a major reservoir of multidrug resistant *A. baumannii*, more precisely MBL producer, and a source of healthcare-associated infections, the study of Gildas et al. (23) supported this statement, by showing that the rate of IMP resistance in *A. baumannii* isolated is 75.6%, in which MBL strains (33.3%), primarily originated from the critical care unit.

Its persistence on abiotic surfaces qualified these strains in ‘hypervirulent’ forms of study conducted by Baba Ahmed-Kazi Tani and Arlet (24) demonstrated that Algeria is one of the countries where carbapenemase-producing Gram-negative bacteria are a major issue. Additionally, other studies in Algeria’s northern regions have looked at the rise of carbapenem-resistant bacteria in hospitals (25). As it has been shown in our study the carbapenem resistance is mediated mainly by the production of carbapenemase class B (MBL), comparable results are reported by Zenati et al. (26). Among 67 *A. baumannii*, 61 isolates were resistant to IMP with MIC greater than 32 μg/mL, in which 32 strains were MBL secretor.

In our study, the interpretation of biofilm results have highlighted that majority *of A. baumannii*, 14 environmental (56%) and 15 clinical (50%) isolates have a moderate to high potential for biofilm formation, as these strains are multidrug resistant either metallo-beta lactamase producer, their persistence on ICU settings will be a serious problem for patients and healthcare workers, which will increases the rate of transmission and re-infection. McQueary and Actis (27) demonstrated that the way diverse clinical strains interact with abiotic surfaces varies depending on the specific properties of both the surface and strain. Moreover, Thompson et al. (28) report that in a murine wound model, *A. baumannii* multidrug resistant strain formed robust biofilms within and above the wound bed on the occlusive dressing.

Yang et al. (29) considered there is a proven correlation between the capacity of *Acinetobacter* isolates to produce biofilms and multi-drug resistance, biofilm producers’ isolates were shown to be resistant to different classes of antibiotics regularly.

Due to the common predominant resistor type (resistotype I) between clinical and environmental isolates, the strains show a significant phenotypic similarity which can lead to a clonal relationship between the two types of isolates. Likewise, comparative studies between *A. baumannii* strains isolated from clinical samples and different surfaces in the ICU prove that they are genetically related (16).

The limitation of our study was that several antimicrobials were not tested to determine extensively drug-resistant potential for *A. baumannii* isolates.

## Conclusion

This study is the first comparison of resistance patterns between clinical and hospital environmental strains isolated from the ICUs in this hospital. A significant similarity level of the antimicrobial susceptibility profiles and biofilm forming potential for environmental and clinical *A. baumannii* isolates, this clonal relationship that has been observed is alarming. Therefore, these results pointed to the ICU as a potential source of multidrug-resistant *A. baumannii* persistence and as a fount of their spread in the ICU settings, engendering cross-contamination and re-infection could easily result in severe outbreaks. Thus, a strict surveillance and infection control measures is required.

## Figures and Tables

**Table 1:** Distribution of environmental and clinical isolated *A. baumannii* from ICU

Clinical samples	Number	%	Environmental samples	Number	%
Wound	1	(3.33)	Beds	15	(60)
Surgical wound	22	(73.3)	Serum stands	4	(16)
Pus	1	(3.33)	Trolleys	1	(4)
Blood	2	(6.66)	Tables	3	(12)
Urine	2	(6.66)	Doors	1	(4)
Catheter	2	(6.66)	Scopes	1	(4)

**Table 2:** Resistotype distribution of environmental and clinical isolated *A. baumannii*

Resistotypes	Antimicrobial resistance profil	Clinical isolates *n* (%)	Environmental isolates *n* (%)
I	AMK/CAZ/GEN/IMP/LVX/MEM/PIP/SXT/TCC/TOB	22 (73)	16 (64)
II	CAZ/GEN/IMP/LVX/MEM/PIP/SXT/TCC/TOB	3 (10)	9 (46)
III	CAZ/GEN/IMP/MEM/PIP/SXT/TCC/TOB	1 (3.33)	0
IV	CAZ/GEN/PIP/SXT/TCC/TOB	2 (6.66)	0
V	AMK/CAZ/GEN/IMP/MEM/PIP/SXT/TCC/TOB	1 (3.33)	0
VI	AMK/CAZ/GEN/IMP/LVX/MEM/PIP/TCC/TOB	1 (3.33)	0

Notes: AMK = amikacin; CAZ = ceftazidime; GEN = gentamicin; IMP = imipenem; LVX = levofloxacin; MEM = meropenem; PIP = piperacillin; SXT = sulfamethoxazole/trimethoprim; TCC = ticarcillin + clavulanic acid; TOB = tobramycin

**Table 3:** Resistance profile of clinical and environmental *A. baumannii*

Antimicrobial	Clinical susceptibility *n* (%)	Environmental susceptibility *n* (%)	*P*-value^a^
Amikacin	24 (80)	16 (64)	0.09
Ceftazidime	29 (96.7)	25 (100)	0.18
Gentamicin	30 (100)	25 (100)	
Imipenem	28 (93.3)	25 (100)	0.09
Levofloxacin	26 (86.7)	21 (84)	0.39
Meropenem	28 (93.3)	25 (100)	0.09
Piperacillin	30 (100)	25 (100)	
Sulfamethoxazole/trimethoprim	28 (93.3)	25 (100)	0.09
Ticarcillin + clavulanic acid	30 (100)	25 (100)	
Tobramycin	30 (100)	25 (100)	

Note:

a Student’s *t*-test
